# Peptides Interfering 3A Protein Dimerization Decrease FMDV Multiplication

**DOI:** 10.1371/journal.pone.0141415

**Published:** 2015-10-27

**Authors:** Mónica González-Magaldi, Ángela Vázquez-Calvo, Beatriz G. de la Torre, Javier Valle, David Andreu, Francisco Sobrino

**Affiliations:** 1 Centro de Biología Molecular Severo Ochoa (CSIC-UAM), Cantoblanco, Madrid, Spain; 2 Departament de Ciències Experimentals i de la Salut, Universitat Pompeu Fabra, Barcelona, Spain; Wuhan University, CHINA

## Abstract

Nonstructural protein 3A is involved in relevant functions in foot-and-mouth disease virus (FMDV) replication. FMDV 3A can form homodimers and preservation of the two hydrophobic α-helices (α1 and α2) that stabilize the dimer interface is essential for virus replication. In this work, small peptides mimicking residues involved in the dimer interface were used to interfere with dimerization and thus gain insight on its biological function. The dimer interface peptides α1, α2 and that spanning the two hydrophobic α-helices, α12, impaired in vitro dimer formation of a peptide containing the two α-helices, this effect being higher with peptide α12. To assess the effect of dimer inhibition in cultured cells, the interfering peptides were N-terminally fused to a heptaarginine (R_7_) sequence to favor their intracellular translocation. Thus, when fused to R_7_, interference peptides (100 μM) were able to inhibit dimerization of transiently expressed 3A, the higher inhibitions being found with peptides α1 and α12. The 3A dimerization impairment exerted by the peptides correlated with significant, specific reductions in the viral yield recovered from peptide-treated FMDV infected cells. In this case, α2 was the only peptide producing significant reductions at concentrations lower than 100 μM. Thus, dimer interface peptides constitute a tool to understand the structure-function relationship of this viral protein and point to 3A dimerization as a potential antiviral target.

## Introduction

Foot-and-mouth disease virus (FMDV) is the etiological agent of the livestock disease that causes the most severe economic losses in endemic areas, and whose reintroduction poses a threat for disease-free countries. The FMDV particle encloses a plus stranded RNA genome of about 8500 nucleotides that contains an open reading frame coding for four structural and nine non-structural mature proteins, flanked by non-coding regions at the 3´and 5´ ends.

Non-structural protein 3A plays important roles in virus replication, virulence and host range [1–3]. This 153-amino acid protein has a conserved N-terminal and a variable C-terminal region in which several deletions and substitutions have been described to affect viral pathogenesis and virulence [4–6]. A membrane topology of the complete 3A protein has been proposed in which the hydrophobic domain spanning residues 59–76 interacts with cellular membranes leaving the N- and the C-termini of the molecule towards the cytosol [7]. A molecular model of the N-terminal fragment, derived from the structure reported for poliovirus (PV) 3A [8], predicted a hydrophobic interface composed of two α-helices spanning residues 25 to 44 as the main determinant for 3A dimerization. In FMDV, 3A homodimerization was evidenced by an *in situ* protein fluorescent ligation assay (PLA) [9]. Replacements L38E and L41E, involving negative charge acquisition at residues predicted to contribute to the hydrophobic interface, reduced dimerization and led to production of infective viruses where the mutated acidic (E) residues reverted to non-polar ones, indicating that preservation of the hydrophobic interface is essential for virus replication. In that same study a peptide reproducing the N-terminal domain of 3A of FMDV (residues I1 to F52, isolate C-S8c1), was shown by Western blotting and mass staining to migrate in both monomeric and dimeric forms, reproducing the dimerization observed in transiently expressed 3A and in infected cells.

Based on these results, we have explored the potential of small dimer interface peptides spanning this region to interfere with in vitro 3A dimerization. Since short peptides do not easily penetrate cells, the interfering peptides were fused to a cell penetrating peptide (CPP) sequence to ensure cellular uptake and eventually an effect on 3A dimer formation and FMDV multiplication.

CPPs are short-to-midsize peptides (5–40 residues), usually cationic, derived from natural sources or synthetically designed, with the ability to pass through cell membranes [10–13] and successfully deliver cargos such as proteins, nucleic acids, small molecule therapeutics and quantum dots, both *in vivo* and *in vitro* [14]. Poly-arginine oligomers are among the best known CPPs, with translocation pathways similar to those of the HIV-1 Tat peptide [15–17]. Accordingly, a heptaarginine (R_7_) CPP sequence was fused N-terminal to dimerization-interfering peptide candidates to ensure their delivery to susceptible cells.

## Materials and Methods

### Peptide design and synthesis

The N-terminal domain (residues 1–52) of protein 3A was assembled by solid phase synthesis at 0.05-mmol scale on Rink-amide ChemMatrix resin (Iris Biotech). After deprotection and cleavage from the resin, the peptide was purified by preparative HPLC using a linear gradient of acetonitrile into water (both +0.1% TFA). Fractions of adequate homogeneity and the expected mass (LC-MS 2010EV, Shimadzu) were used for biological assays. The partial α1 (residues 25–33), α2 (37–44) and α12 (25–44) sequences, their R7-elongated (see Table 1 for details) derivatives, as well as the octa-arginine control, were synthesized by similar protocols on Rink-amide MBHA resin (Iris Biotech). Fluorescent versions of the peptides were made by coupling 5(6)-carboxyfluorescein to the N-terminus.

**Table 1:** Synthetic peptides used in this study^a^.

			Mass	
Peptide^b^	3A residues	Sequence^b^	monoisotopic	found
N-terminal	(1–52)	ISIPSQKSVLYFLIEKGQHEAAIEFFEGMVHDSIKEELRPLIQQTSFVKRAF	6036.0074	6036.563
α1	(25–33)	FFEGMVHDS	1066.4542	1067.283
α2	(37–44)	ELRPLIQQ	994.5924	995.924
α12	(25–44)	FFEGMVHDSIKEELRPLIQQ	2414.2417	2415.972
R_7_-α1	(25–33)	RRRRRRRXFFEGMVHDS	2272.2461	2273.995
R_7_-α2	(37–44)	RRRRRRRXELRPLIQQ	2200.3842	2201.502
R_7_-α12	(25–44)	RRRRRRRXFFEGMVHDSIKEELRPLIQQ	3620.0335	3621.505
Flu-α1	(25–33)	Flu-FFEGMVHDS	1424.5020	1425.542
Flu-α2	(37–44)	Flu-ELRPLIQQ	1352.6401	1353.505
Flu-α12	(25–44)	Flu-FFEGMVHDSIKEELRPLIQQ	2772.2894	2774.500
Flu- R_7_-α1	(25–33)	Flu-RRRRRRRXFFEGMVHDS	2630.2938	2631.063
Flu- R_7_-α2	(37–44)	Flu-RRRRRRRXELRPLIQQ	2558.4319	2559.887
Flu- R_7_-α12	(25–44)	Flu-RRRRRRRXFFEGMVHDSIKEELRPLIQQ	3978.0812	3980.426
R8	control	RRRRRRRR	1266.5304	1267.219

^a^ All peptides are C-terminal carboxamides

^b^ Flu = fluoresceine carboxylic acid; X = 6-aminohexanoic acid (spacer)

### Cells, viruses and antibodies

The origin of BHK-21 cells and culture procedures has been described [18]. A viral stock from type C FMDV C-S8c1 isolate [19] was produced by amplification in BHK-21 cells. A bovine enterovirus (BEV) [20] isolate was used as control for virus specificity. Monoclonal antibodies (Abs) against the FMDV 3A (2C2), VP1 (SD6) and rabbit polyclonal antibodies to 3A (479) and β-tubulin were employed [7, 9, 21].

### Infection and virus titration

Cells were infected with FMDV at the multiplicity of infection (moi) indicated. After 60 min adsorption, the viral inoculum was removed, cell monolayers washed twice with DMEM and fresh medium containing 1% FBS was added; this time point was considered 0 h post-infection (p.i). Virus titration in semisolid agar medium was as described [9].

### Dimer interference assay and in situ protein ligation assay (PLA)

For in vitro interference, 16.5 μm of the N-terminal (1–51) domain was incubated with different molar ratios of interfering peptides (α1, α2, α12) and R_8_ negative control, at room temperature for 30 min. Next, sample buffer was added, peptides were resolved by 12% SDS-PAGE and their migration monitored by Coomasie blue staining and western blotting. For in vivo interference, BHK-21 cells were grown on glass cover slips and incubated with 100 μM of either α1, α2 or α12 R_7_-fused peptides for 1 h at 37°C. 24 h later, cells were transfected with 1 μg of pcDNA3A using Lipofectamine 2000 (Life Technologies, Alcobendas, Spain). At 24 h post–transfection (pt) monolayers were fixed in 4% paraformaldehyde and permeabilized as described [7]. Primary Ab 2C2 was prepared using the Probemaker kit (OLINK, Bioscience) to generate plus and minus PLA probes. Then, cells were incubated with conjugated primary Abs and the signal development (ligation, amplification and hybridization) was performed as recommended the manufacturer. Finally, samples were further incubated with primary polyclonal Ab 346 and anti-rabbit IgG secondary Abs coupled to Alexa Fluor (AF) 647 (Life Technologies) to detect 3A protein. Slides were mounted with a cover slip using a minimal volume of Duolink II mounting medium with DAPI. Cells were observed with a Confocal LSM710 Vertical (Carl Zeiss Iberia, Tres Cantos, Spain) microscope. As reported for dimerization detection by PLA [22], fluorescence was quantified using the ImageJ software (analyze particles plug-in), n≥10.

For interference with virus, BHK-21 cells grown overnight were treated with different concentrations of R_7_-fused peptides (α1, α2, α12) and R_8_ for 1 h at 37°C. Fresh medium was added and after 24 h cells were infected with the corresponding virus and frozen at 8 h pi. For virus titration, infected cells were subjected to three freeze-thaw cycles, and the total (intracellular and medium-released) virus yield was determined by plaque assay in BHK-21 cells as described [18].

### Western blot analysis

BHK-21 cells incubated with peptides and infected were collected and processed as in [7]. Briefly, equal volumes of each sample were loaded on a SDS-PAGE 12%, transferred onto a nitrocellulose membrane and the proteins detected by incubation with the selected primary antibody and the corresponding horseradish peroxidase-coupled secondary antibody that was developed using a chemiluminescence kit (Perkin-Elmer).

### Cell viability test

The effect of CPPs on cell viability was determined using the CellTiter-Glo Luminiscent Cell Viability assay (Promega). BHK-21 cells were seeded in 6-well plates and incubated with increasing (0–100 μM) CPP concentrations for 24 h, then assayed as recommended by the manufacturer.

### R_7_-fused peptide penetration

Cells were grown on glass cover slips overnight and treated with 10 μM fluorescein-labelled peptides for 30 min. Subsequently cells were fixed in 4% paraformaldehyde for 15 min at room temperature, blocked and permeabilized as described [7]. Samples were incubated with phalloidin–tetramethylrhodamine B isothiocyanate (TRITC; Sigma) for 1 h to stain actin filaments for light microscopy and To-Pro-3 (Life Technologies) was used as a nuclear counterstain. Finally, samples were mounted in prolong gold antifade (Life Technologies) and cells were observed with a Microradiance confocal (Carl Zeiss) microscope.

### Data analysis

To probe statistical significance of the data, one-way analysis of the variance was performed with the SPSS 21.0 statistical package (IBM; Armonk, NY) for Windows. For multiple comparisons, Bonferroni's correction was applied. The data are presented as means ± the standard deviations and statistically significant differences are indicated in the figures by an *.

## Results

### Peptide design and synthesis

**Fig 1:**
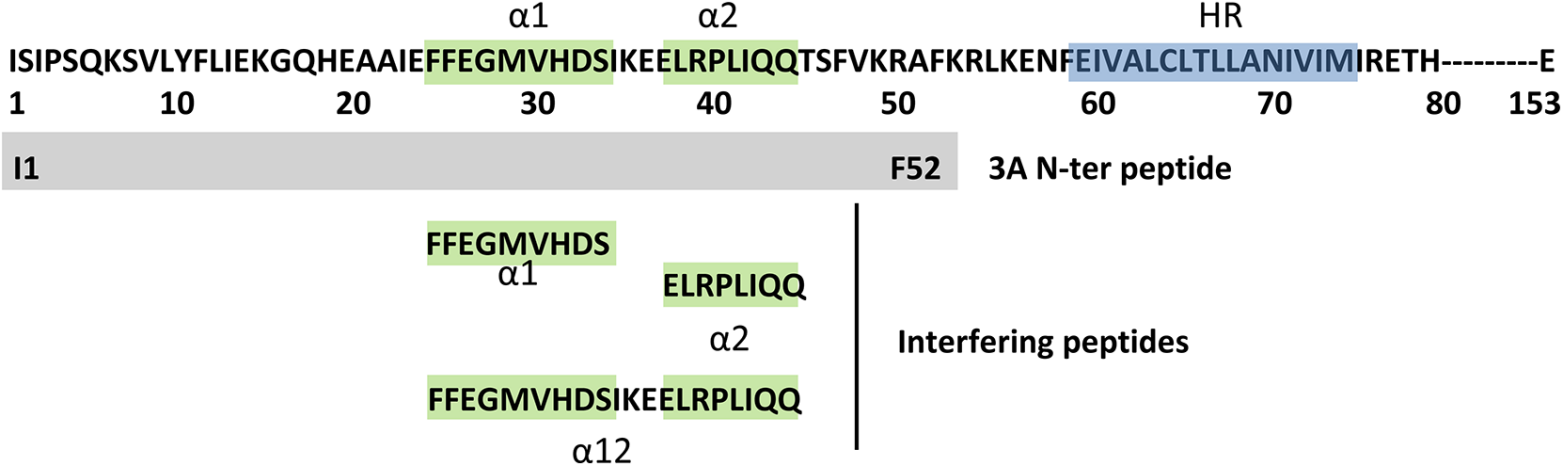
Amino acid sequence (residues 1–153) of FMDV protein 3A. The two α-helices are represented in green and the hydrophobic region (HR) of the protein in blue. Peptides used in this work are shown: N-ter (I1-F52) and three interfering peptides: α1(F25-S33), α2(E37-Q44) and α12(F25-Q44).

Structural prediction of the N-terminus of 3A posits two alpha helices that mediate protein homodimerization. Replacements at L38E and L41E reduced the dimerization signal in a protein ligation assay and prevented detection of dimer species in transiently expressed 3A (Gonzalez-Magaldi et al., 2012). To explore this evidence further, we designed three peptides spanning helices α1 (residues 25–33), α2 (37–44) or both α12 (25–44) with a view to interfere with 3A dimerization (Fig 1). To ensure efficient intracellular delivery, each interfering sequence was extended at the N-terminus with seven Arg (R_7_) residues conferring cell penetrating peptide (CPP) properties (R_7_- fused peptides) followed by a 6-aminohexanoic acid flexible spacer residue. Fluorescein-labeled versions of all peptides (with or without R_7_) were also made (Table 1). All peptides were obtained with high (>95% HPLC) purity by Fmoc solid phase synthesis methods. In addition, octa-arginine (R_8_) used as negative control and the entire N-terminal domain (residues 1–53) used as a dimerization model in vitro, were produced.

### CPP-fused peptides penetrate cells

**Fig 2:**
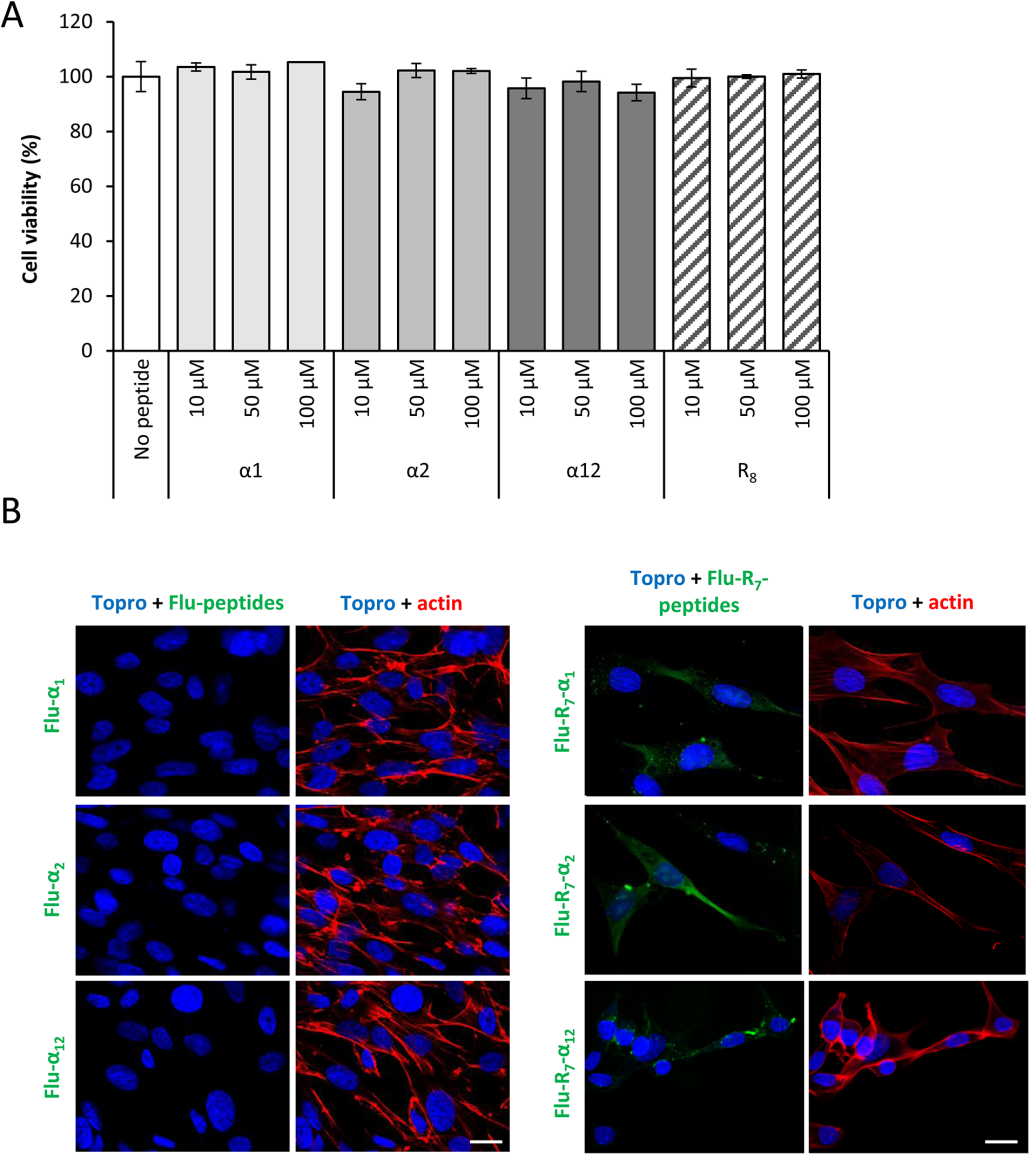
CPP-fused peptides penetrate cells. (A) Effect of R_7_-elongated peptides on cell viability was determined by ATP measurement with CellTiter-Glo^®^ luminescent cell viability assay. (B) BHK-21 cells were incubated with 10 μM of labeled peptides fused (Flu-R_7_-α1, Flu-R_7_-α2, Flu-R_7_-α12) or not (Flu-α1, Flu-α2, Flu-α12) with R_7_ for 30 min at 37°C. Then, the samples were processed for confocal microscope analysis using phalloidin-TRITC (red) and To-Pro 3 (blue) to stain actin filaments and nuclei, respectively. Bar: 20 μm

We first evaluated the effect of peptides on cell viability. None of the R_7_-fused peptides, in the 10–100 μM concentration range examined, had noticeable effects on BHK-21 cell viability after 24 h treatment (Fig 2A). To evaluate the penetrating activity of R_7_-peptides we examined the cellular uptake of their fluorescein (Flu)-labeled versions by confocal microscopy. Flu-labeled α1, α2 and α12 lacking the R_7_ motifs were used as controls. Phaloidin was used to visualize the cortical actin in order to make sure the green peptide fluorescent dots were inside the cell. Data showed (Fig 2B) that R_7_-fused peptides were readily internalized into BHK-21 cells while peptides devoid of the CPP moiety were not.

### Peptides enhance 3A dimer dissociation

**Fig 3:**
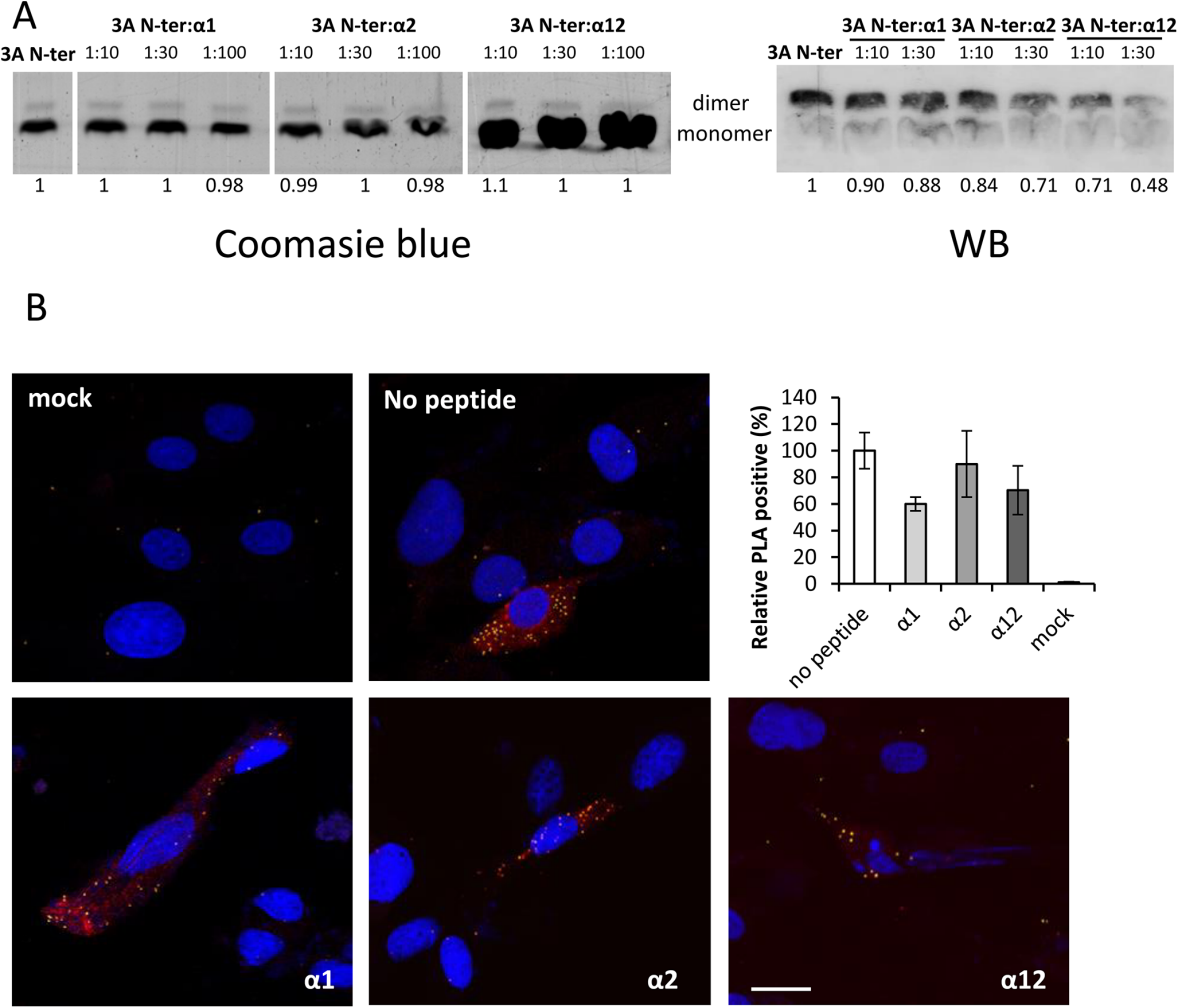
Peptides induce dimer dissociation. (A) A fixed concentration of 3A N-ter peptide was incubated with different ratios of interfering peptides lacking R7 (α1, α2, α12) at room temperature for 30 min. The samples were resolved on two 12% SDS-PAGE parallel gels, one of which was stained with Coomassie blue, the other transferred to nitrocellulose membrane and blotted with Ab 479. Bands intensities of dimer form of peptide N-ter were quantified and expressed relative to that control of control sample (N-ter peptide alone). (B) BHK-21 cells were incubated with each R_7_-fused peptide (100 μM) and 24 h later were transfected with pcDNA3A. Samples were next submitted to in situ PLA using a 3A-specific monoclonal Ab (2C2) coupled to two oligonucleotide probes to assess 3A homodimerization. A negative control (mock) of non-transfected cells was included. Nuclei were stained using DAPI (blue). Graphs represent percentage of dots relative to that of cells incubated with no peptide. Raw data were quantified using the ImageJ software (analyze particle plug in) n≥10. Standard errors are represented.

Different in vitro assays were designed to assess the effect of peptides on 3A dimerization. Synthetic N-terminal (1–52) peptide was first used as a dimerization model, as described [9]. After incubating with the interfering peptides lacking R_7_ (α1, α2 or α12), we analyzed dimerization by coomasie blue staining (Fig 3A). In this assay, peptides α1 and α2 showed a rapid migration and were not observed in the gels. On the other hand, the increase in the monomer band observed for lower N-ter: α12 ratios reflected the accumulation of N-ter and α12 peptides whose electrophoretic migration was similar. No clear differences were found in the intensity of the dimer band observed when N-ter peptide was incubated with the interfering peptides, relative to the N-ter peptide alone (Fig 3A). When dimerization was analyzed by immunoblotting with polyclonal antibody 479, the only antibody available that recognized peptide N-ter, a decrease in the intensity of the dimer band, relative to that of N-ter alone, was observed with the three interfering peptides, being the effect higher for α12 (Fig 3A). In this case, detection of the expected increase in the monomer band was impaired by the higher amount of monomer as well as by the fact that antibody 479 mainly recognized the dimer, as reported [9].

Next, the ability of R_7_-fused peptides α1, α2 or α12 to interfere with dimerization of transiently expressed 3A in BHK-21 cells was assayed. For that purpose, cells were incubated with the peptides, and 24 h later were transfected with pcDNA3A. To assess 3A dimerization, in situ PLA with a 3A-specific monoclonal Ab (2C2) was used (Fig 3B). The number of fluorescent dots in transfected cells (expressing 3A protein) incubated with no peptide was taken as 100% dimerization, and those in peptide-incubated cells were related to that value (Fig 3B). A decreasing trend in 3A dimerization was observed for R_7-_fused peptides α1 and α12. The lack of effect of peptide α2 in the dimerization of N-ter peptide (Fig 3A) could be due, among other factors, to differences in the accessibility of α2 region in the in situ 3A PLA relative to the cell free peptide dimerization assay.

### R_7_-fused peptides inhibit FMDV production

**Fig 4:**
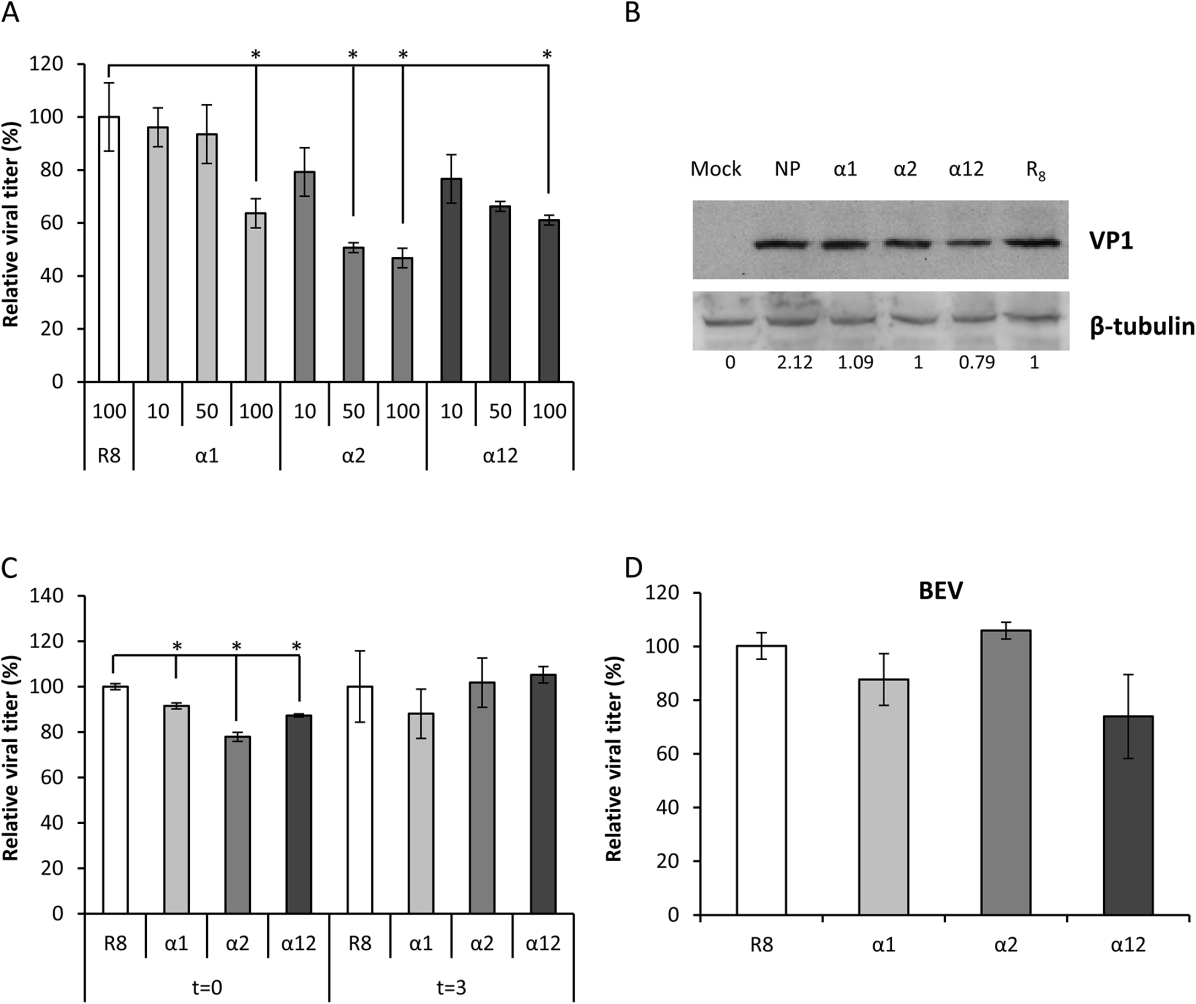
R_7_-fused peptides inhibit FMDV production in BHK-21 cells. (A) Cells were incubated with different concentrations of R_7_-fused peptides (α1, α2 and α12) for 1h at 37°C; 24 h later cells were infected with C-S8c1 (moi of 1) and 8 h later the total viral titer was determined by plaque assay. Cells treated with octaarginine (R_8_) were used as negative control. (B) Cells treated and infected as in (A). At 5 h p.i., cells were lysed and processed by western blot using Ab SD6 to VP1 and 193 to β-tubulin. Quantitative densitometry of VP1 protein expression normalized for β-tubulin expression, and relative to that of R8-treated infected cells, is indicated. (C) Cells were incubated for 1 h at 37°C with the different R_7_-fused peptides (100 μM) at the time of viral infection (t = 0) or at 3 h p.i. (t = 3) with a moi of 1; 8 h later total viral titer was determined by plaque assay. (D) Cells were incubated with 100 μM of different R_7_-fused peptides as before, 24 h later were infected with BEV (moi of 1) and 8 h pi the total viral titer was determined by plaque assay. Bars represent the mean percentage of treated and infected cells ± SD, normalized to the level of infection of cells no peptide treated. Statistically significant differences between untreated cells or peptide treated cells are indicated by an asterisk (ANOVA P≤0.05).

FMDV-susceptible BHK-21 cells were treated with increasing concentrations of R_7_- fused peptides 24 h prior to FMDV infection (moi 1) and total viral production was titrated in BHK-21 cells. Relative to R_8_-treated control cells, the cells treated with R_7_-fused peptides α1, α2 and α12 showed a dose dependent inhibition of viral production (Fig 4A). The highest inhibition (ca. 50%) was found for peptide α2 at 100 μM. When the level of virus protein synthesis in infected cells previously incubated with R_7_-fused peptides was quantified a decrease of VP1 protein was only observed for peptide α12 (Fig 4B). The reasons behind the differences in the effect of the interfering peptides on the reduction of the viral titer and the synthesis of VP1 protein remain to be determined.

When peptides were added at infection time, reductions, albeit of lower magnitude than those observed at 24 h prior to infection were found, while no reductions were observed when peptides were added 3 h p.i (Fig 4C). This result suggests that internalization of the interfering peptides as to reach cell locations in which effectively interfere with virus replication is a time-dependent process and that early steps of viral infection are more susceptible to peptide interference than latter infection stages.

The specificity of the inhibition was confirmed by infecting peptide treated cells with bovine enterovirus (BEV), a different picornavirus. Results in Fig 4D indicate that none of the peptides exerted significant inhibitions on BEV growth.

## Discussion

The formation of a protein dimer is a process that generally responds to a specific protein function or is a consequence of another protein interaction. There are many nonstructural proteins of different picornaviruses [23–25], including FMDV [26] that are described to dimerize/multimerize. Recent structural and biophysical studies show that protein dimerization or oligomerization is a key factor in the regulation of different protein functions [27], including proteins relevant for virus replication [28]. FMDV nonstructural protein 3A is involved in relevant functions in virus replication [1, 29]. It shares common features with proteins from other picornaviruses, albeit with structural and functional differences such as a longer amino acid sequence and insensitivity to the Golgi disrupter brefeldin A [30, 31]. In common with poliovirus and coxsackievirus, FMDV 3A can form homodimers whose biological function is not well understood, although it could be related to the multimerization of other non-structural proteins of different picornaviruses [26, 32].

Inhibition of viral proteins by molecules or small synthetic peptides is studying as antiviral strategy few years ago [33, 34]. Moreover, small peptides mimicking residues involved in the dimer interface have been used to interfere with dimerization and thus gain insight on its biological function [35]. Here, we intended to interfere with 3A dimerization and to analyze its effect on viral replication, hence viral production. In this work, we show that such dimer interface peptides can impair in a dose dependent manner in vitro dimer formation of a peptide containing the two α-helices that make up the 3A dimer interface. This effect was higher with peptide α12, spanning the two hydrophobic α-helices. Dimer inhibition was also observed in cells transiently expressing the complete 3A protein. To this end, the interfering peptides α1 and α12 were N-terminally fused to a heptaarginine (R_7_) sequence, a well-known CPP motif that favors intracellular translocation [15, 17]. Thus, when fused to R_7_, interference peptides (100 μM) were able to inhibit in situ dimerization of transiently expressed 3A, the higher inhibitions being found with R_7_-fused peptides α1 and α12. The 3A protein dimerization impairment exerted by the peptides correlated with significant reductions in the viral yield recovered from peptide-treated FMDV infected cells. In this case, α2 was the only R_7_-fused peptide producing significant reductions at concentrations lower than 100 μM. The discrepancies observed in the inhibitions exerted by α2 in in situ dimer formation (Fig 3B) and viral yield (Fig 4A) could reflect differences in the amount, time-course and interaction with cell components between transiently expressed 3A and that synthesized in the context of virus infection in a manner accomplished with the other virus proteins. The virus yield reduction observed was specific for FMDV as no significant effect was observed in peptide-treated cells infected with BEV.

Taken together, the above findings show the feasibility of inhibiting FMDV 3A dimerization by means of peptides reproducing the dimer interface, and the effect of this inhibition on virus multiplication, providing with a tool to understand the structure-function relationship of this viral protein and pointing to 3A dimerization as a potential antiviral target.
